# Effectiveness of the prevention of mother-to-child transmission of HIV protocol applied at Saint Camille Medical Centre in Ouagadougou, Burkina Faso

**DOI:** 10.7448/IAS.17.4.19701

**Published:** 2014-11-02

**Authors:** Serge Theophile Soubeiga, Cyrille Bisseye, Rebecca Compaore, Elsa Assengone, Djeneba Ouermi, Florencia Djigma, Tani Sagna, Virginio Pietra, Jacques Simpore

**Affiliations:** 1 Molecular Biology, University of Ouagadougou/CERBA/LABIOGENE, Ouagadougou, Burkina Faso; 2 Faculty of Sciences, Department of Biology, University of Sciences and Technology of Masuku, Franceville, Gabon; 3 Biology, University of Brescia, Brescia, Italy

## Abstract

**Introduction:**

Despite many prevention efforts, the number of children infected by HIV in sub-Saharan Africa through vertical transmission remains high. This infection can be reduced through programmes of prevention of mother-to-child HIV transmission (PMTCT). The objective of this study was to evaluate the effectiveness of the PMTCT protocol at Saint Camille Medical Centre in Ouagadougou, Burkina Faso.

**Methods:**

From August 2012 to September 2013, samples of dried blood spot (DBS) were collected from 160 children aged 6 weeks born to HIV-1 positive mothers who were under PMTCT protocol at Saint Camille Medical Centre and 40 children of the same age group from orphanages and whose mothers were dead or unknown. The samples were tested using the Abbott Real Time HIV-1 Qualitative kit. The clinical data of mothers were collected and analyzed using SPSS Version 17.0 and Epi Info Version 6.0 softwares.

**Results:**

Among pregnant women in this study, 52.5% were predominantly young (24–29 years) and 60.62% were housewives. In total, 50.5% (101/200) were in combination of AZT/3TC/NVP and 29.5% (59/200) were on prophylaxis (AZT/3TC). The rate of vertical transmission of HIV-1 was 0.0% (p<0.001) in children whose mothers were taking a combination of AZT/3TC/NVP (0/101) or were on a prophylaxis AZT/3TC treatment (0 /59). The rate of HIV-1 transmission in orphaned children was 15.0% (6/40).

**Conclusions:**

The PMTCT protocol is effective and reduces very significantly (p<0.001) the risk of transmission of HIV-1 from mother to child. In addition, screening by PCR of orphaned children vertically infected with HIV, enabled them to receive an early treatment.

